# *In silico* identification and functional validation of allele-dependent AR enhancers

**DOI:** 10.18632/oncotarget.3019

**Published:** 2015-02-27

**Authors:** Sonia Garritano, Alessandro Romanel, Yari Ciribilli, Alessandra Bisio, Antoneta Gavoci, Alberto Inga, Francesca Demichelis

**Affiliations:** ^1^ Laboratory of Computational Oncology, CIBIO, Centre for Integrative Biology, University of Trento, Italy; ^2^ Laboratory of Transcriptional Networks, CIBIO, Centre for Integrative Biology, University of Trento, Italy; ^3^ HRH Prince Alwaleed Bin Talal Bin Abdulaziz Alsaud Institute for Computational Biomedicine, Weill Medical College of Cornell University, New York, NY, USA; ^4^ Institute for Precision Medicine, Weill Medical College of Cornell University and New York Presbyterian Hospital, New York, NY, USA

**Keywords:** Androgen Receptor (AR), polymorphic regulatory regions, enhancer, allele-specific, Estrogen Receptor (ER)

## Abstract

Androgen Receptor (AR) and Estrogen Receptors (ERs) are key nuclear receptors that can cooperate in orchestrating gene expression programs in multiple tissues and diseases, targeting binding elements in promoters and distant enhancers. We report the unbiased identification of enhancer elements bound by AR and ER-α whose activity can be allele-specific depending on the status of nearby Single Nucleotide Polymorphisms (SNP). ENCODE data were computationally mined to nominate genomic loci with: (i) chromatin signature of enhancer activity from activation histone marks, (ii) binding evidence by AR and ER-α, (iii) presence of a SNP. Forty-one loci were identified and two, on 1q21.3 and 13q34, selected for characterization by gene reporter, Chromatin immunoprecipitation (ChIP) and RT-qPCR assays in breast (MCF7) and prostate (PC-3) cancer-derived cell lines. We observed allele-specific enhancer activity, responsiveness to ligand-bound AR, and potentially influence on the transcription of closely located genes (*RAB20, ING1, ARHGEF7, ADAM15*). The 1q21.3 variant, rs2242193, showed impact on AR binding in MCF7 cells that are heterozygous for the SNP. Our unbiased genome-wide search proved to be an efficient methodology to discover new functional polymorphic regulatory regions (PRR) potentially acting as risk modifiers in hormone-driven cancers and overall nominated SNPs in PRR across 136 transcription factors.

## INTRODUCTION

Knowledge of transcriptional and chromatin regulators acting at promoter and enhancer elements has increased considerably in the last decade, highlighting a causative role for gene expression deregulation in different diseases. Cancer, neurological disorders, autoimmunity, and cardiovascular disease can be influenced by mutations in regulatory sequences or in transcription factors (TFs), cofactors, and chromatin regulators [1, 2].

Enhancers contain multiple *cis*-elements able to recruit TFs; the type and concentration of available TFs at a specific time and location determine the efficiency of initiation complex formation and RNA polymerase II recruitment. *Cis*-elements can be located quite far from TSS, for example within internal introns, intergenic DNA or even in a different chromosome [3]. Promoter and enhancer need to be spatially close, for example through the formation of loops and other chromatin super-structures [4, 5].

Specific histone modifications have been used to map the presence of enhancers or promoters within a particular genomic region. Methylation and Acetylation of Lysine residues in histone tails are key events. There is strong evidence that the concomitant presence of mono- and tri-methylation of Lysine 4 of histone H3 (H3K4me3) marks transcriptionally active promoters, while mono-methylation, but not tri-methylation, of the same histone residue (H3K4me1) is a marker of enhancers. Moreover, it has been proven that histone H3 Lysine 27 acetylation (H3K27Ac) is able to distinguish active enhancers from inactive or poised enhancer elements containing H3K4me1 alone [6]. These findings are further supported by the enrichment of these epigenetic marks in nucleosome free regions (NFR), p300 binding (a transcriptional co-activator, enzymatically acting as histone acetyl transferase - HAT), and increased nuclease sensitivity [7], all of them markers of open chromatin.

In the last decade, the Encyclopedia of DNA Elements Project (ENCODE) has performed a large number of sequence-based studies to map functional elements across the human genome leading to the biochemical characterization of intronic and intergenic regions [8–10]. The ENCODE results also highlighted cell type specificity of transcriptional regulator binding sites or chromatin states, consistent with the interpretation of noncoding variants relevant to human diseases. Genome-wide association studies (GWAS) have identified more than 150 polymorphic loci associated with increased susceptibility to cancer [11], the majority of which reside outside of known protein-coding sequences potentially influencing the regulation of critical target genes through distal enhancer elements [12–14].

The availability of these annotations opens up to a plethora of *in silico* studies towards the understanding of the role of non-coding inherited in human diseases. So far direct functional implications have been demonstrated only for few of the noncoding SNPs identified through GWAS [15]. To what extent GWAS genetic variants are of clinical or public health importance especially for developing preventive or therapeutic interventions is an open question. The challenge is to demonstrate how single variants or combinations can increment disease susceptibility by perturbing the expression of a transcript, disrupting the function of a protein or affecting regulatory sequences.

We reasoned that ad hoc computational searches of annotated regions combined with genome-wide TF sites from ChIP-Seq experiments and *in vitro* functional assay could identify polymorphisms that likely influence target gene regulation in an allele-dependent manner. As proof of concept, we focused on polymorphisms within regulatory elements bound by two TFs, ER-α and AR, that are key nuclear receptors in common human cancers characterized by a genetic component to their etiology, breast and prostate cancers [16, 17].

Nuclear receptors belong to a large superfamily of evolutionary related TFs that are able to integrate signals coming from outside of the cells and influence gene expression. Glucocorticoid Receptor (GR), Androgen Receptor (AR), Estrogen Receptors (ERs) and Retinoic Acid Receptors (RARs) are among the most important and studied members of the family [18]. Ligand binding causes a conformational change that enables dissociation from the inhibitory complex, homo- or hetero-dimerization, nuclear translocation, DNA binding, recruitment of co-activators, thus stimulating transcription of their target genes [19]. Interestingly, it has been discovered that the recruitment of AR can occur more often at gene-distal and intragenic sites rather than at proximal promoter regions. Deregulation of androgen/AR signaling perturbs the normal development of reproductive tract and accounts for a wide range of pathological conditions such as androgen-insensitive syndrome and prostate cancer [20]. Indeed, most prostate cancers express AR, are androgen-dependent for their growth and, as a result of androgen withdrawal, can undergo either cell cycle arrest or even apoptosis. For these reasons, androgen deprivation therapy (ADT) is an effective treatment in prostate cancer, although most patients progress to castration-resistant prostate cancer with an increase of AR expression levels and hypersensitivity to androgen-based therapies. Estrogen Receptor α and β are sequence-specific TFs that play important roles in development as well as in physiological or pathological conditions in somatic cells, able to influence transcription once activated through the binding to estrogenic compounds ligands. Deregulation of ERs, particularly ER-α, has been extensively studied and associated with cancer development. ER-α induces cell growth and proliferation even if its expression in tumor correlates with a favorable prognosis in endocrine therapy [21, 22].

The broad coverage of the ENCODE annotations allows for the robust investigation of the impact that both somatic and germ line single nucleotide variants can have on distal *cis*-regulatory sequences [23]. Through a genome wide methodologically unbiased approach applied across multiple cell lines, we identified a set of regulatory elements targeted by one or multiple TF spanning (or in proximity) SNPs and named them polymorphic regulatory regions (PRR). *In vitro* validation experiments on selected loci bound by ER-α and by AR indicate that this approach can detect functionally distinct allelic variants acting as AR-responsive distant enhancers.

## RESULTS

### *In silico* detection and characterization of polymorphic regulatory regions

**Figure 1:**
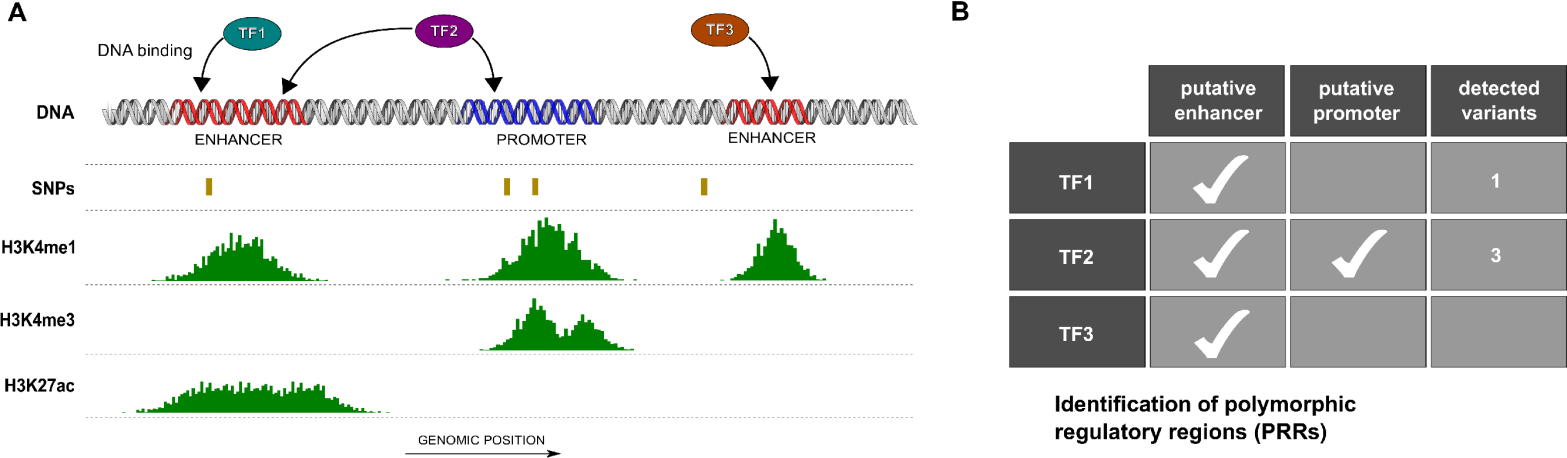
Identification of polymorphic regulatory regions (PRRs) **(A)** Schematic view of putative PRRs study selection. Based on genomic coordinates regulatory regions (e.g. enhancers and promoters) from ENCODE open chromatin and activation histone marks peaks (e.g. H3K4m1, H3K4me3 and H3K27ac), polymorphic sites (SNPs) and transcription factor (TF) binding regions were combined. **(B)** Multiple transcription factors may occupy one or more PRRs characterized by different patterns of polymorphic loci.

*In silico* analyses identified putative regulatory regions bound by one or more TFs and encompassing polymorphic sites as schematically represented in Figure 1A and 1B, here defined as PRRs. Overall we identified PRRs involving 136 transcription factors and focused on AR and ER-α occupancy data. Table 1 lists the number of SNPs within *consensus regulatory regions* bound by either one or both TFs. A total of 591 (553) SNPs within at least one consensus regulatory region and bound by AR (or ER-α) were identified. A subset of 41 SNPs were located within consensus regulatory regions bound both by AR and ER-α, of which 14 SNPs were in regions compatible with enhancer activity and 19 with promoter activity, based on histone tail marks. To select PRRs for downstream validation we further considered SNP allele frequencies, availability of genotype-phenotype data (see Materials and Methods) and SNP genotypes of available cell lines. Two PRRs were chosen for experimental characterization; the first located in 1q21.3 (spanning rs2242193), hereafter referred to as Locus 1 and the second in 13q34 (spanning rs9521825), hereafter referred to as Locus 2 (Figure S1). Table S2 contains the SNP characteristics.

**Table 1:** Number of SNPs (dbSNP138) from the human genome that intersect regulatory regions bound by AR and/or ER-α

	AR	ER-α	AR and ER-α
H3K4me1	525	500	33
H3K4me1+H3K3me3	257	281	19
H3K27ac	396	410	25
H3K9ac	363	437	33
DNase	184	284	11
FAIRE	76	161	4
Union	591	553	41

Supplementary Table S3A and S3B reports the numbers of SNPs that overlap consensus regulatory regions bound by at least one of the 136 TFs in CEU population and in all populations, respectively. In addition, the complete lists of SNP identifiers within consensus regulatory regions bound by every TF included in the study are available online (http://demichelislab.unitn.it/PRRTFSNP).

### The *in silico* selected regions showed enhancer activity

**Figure 2:**
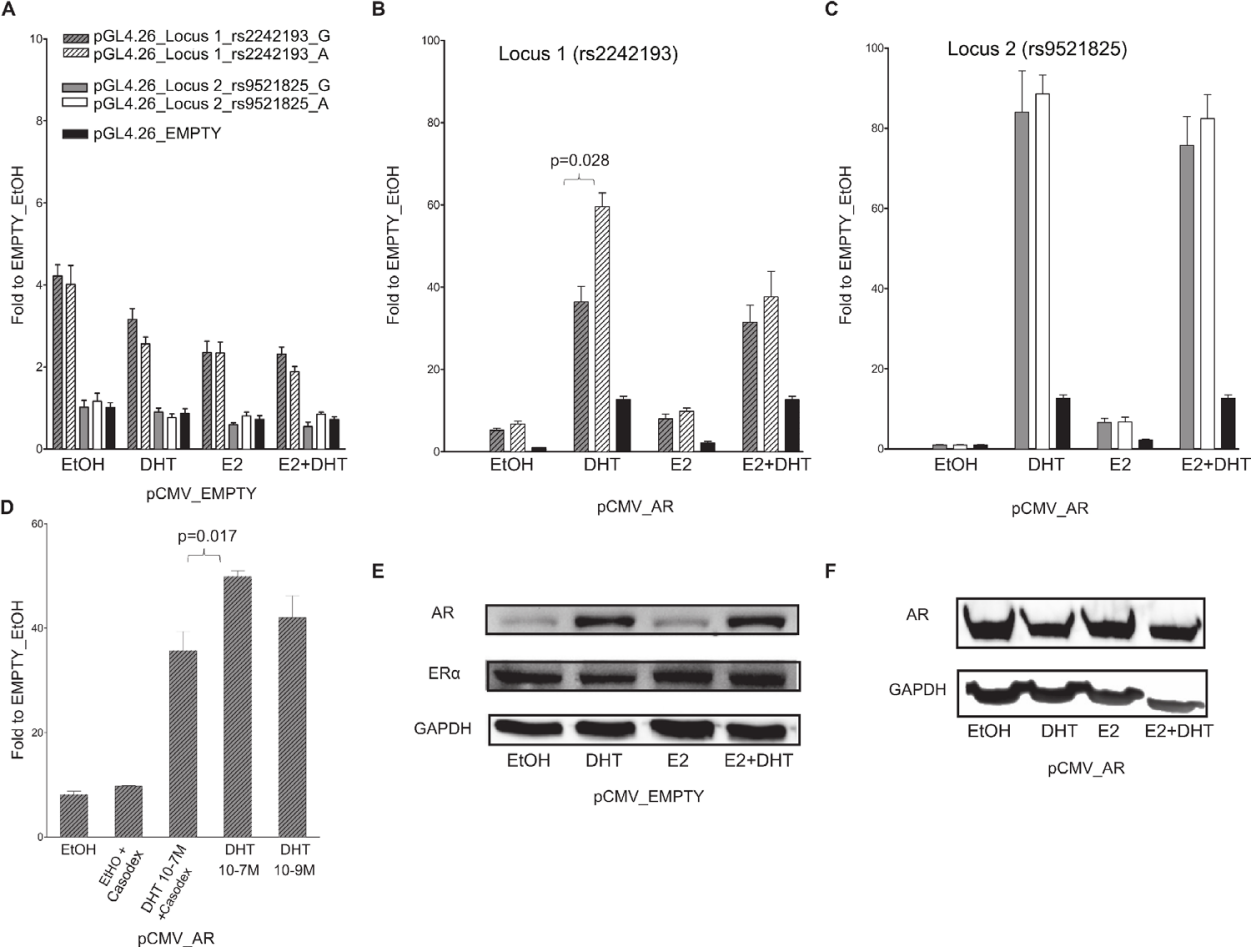
The selected locus 1 and locus 2 regulatory regions act as AR-dependent enhancers in the MCF7 cell line **(A)** MCF7 cells were co-transfected with pCMV_EMPTY vector along with different pGL4.26 reporter constructs containing selected regulatory regions isolated from two putative AR- or ER-target elements (indicated in the legend). Twenty-four hour post-transfection cells were treated for 16 hours with E2, DHT or the combination of the two compounds to stimulate respectively ER- or AR-dependent transcription. **(B – C)** MCF7 cells were co-transfected with pCMV_AR vector along with pGL4.26_Locus 1 or _Locus 2 reporter constructs using the same experimental conditions as in panel A. Wild-type (dashed- or plain-grey bars) and SNPs rs2242193 or rs9521825 (dashed- or plain-white bars) containing constructs were tested. Indicated is the percentage value of statistical relevant differences (Student's t-test). **(D)** AR-dependent effect was determined performing luciferase assays by treating MCF7 cells with two different concentrations of DHT and by adding the AR inhibitor Casodex. All the bars presented in the gene reporter assays represent the averages and the standard deviations of at least three biological replicates each performed in triplicate. **(E)** To evaluate the endogenous cellular levels of AR and ER in MCF7 cells untreated and treated with E2, DHT or the combination of the two compounds, a western blot was performed. GAPDH expression was used as loading control. **(F)** A western blot was carried out as in E to verify the amount of AR protein in MCF7 cells over-expressing AR untreated and treated as indicated.

**Figure 3:**
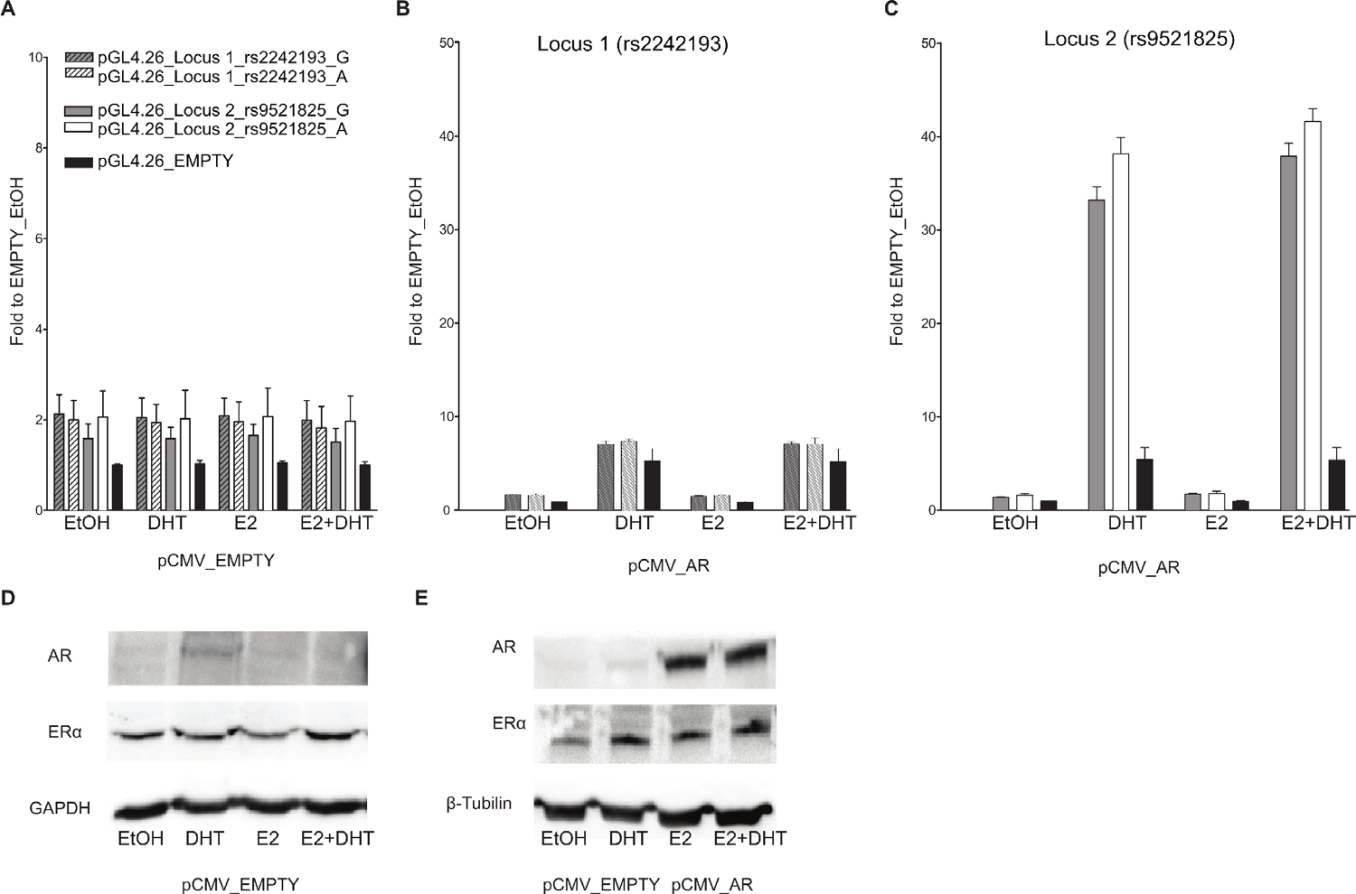
Only locus 2 is AR responsive in PC-3 cells Experiments were performed with the same experimental setting of Figure 2. Gene reporter assays were conducted in PC-3 cells co-transfected with pCMV_EMPTY vector **(A)** or with AR over-expression vector **(B)** along with different the pGL4.26 reporter constructs (wild-type or containing the SNPs rs2242193 or rs9521825) -pGL4.26_Locus 1 (panel **B**), _Locus 2 (panel **C**). **(D)** The amount of ER alpha endogenous protein levels was evaluated through western blot analysis upon treatment with E2, DHT or the combination of the two compounds. **(E)** The same western blot was performed also in PC-3 cells transiently transfected with an empty vector or an expression vector for AR to demonstrate the increase in protein amount and to test the impact of E2 or DHT treatment on AR or ER expression. GAPDH or β-Tubulin endogenous expression was used as loading control.

To address whether the *in silico* selected PRRs exhibit enhancer activity modulated by ER and/or AR, we conducted dual luciferase reporter gene assays in MCF7 and PC-3 cells transiently co-transfected with different pGL4.26 reporter constructs, along with pRL-SV40 control vector. No induction of reporter expression was observed after treatment with the DHT and/or E2 compared to the treatment with solvent (ethanol, EtOH). In MCF7 cells a clear induction (luciferase activity relative to the one obtained with the cells transfected with the pGL4-empty vector) was observed with the pGL4-Locus1 sequence in EtOH condition, demonstrating an intrinsic ligand-independent enhancer activity of this sequence, whereas no statistically significant induction was observed in PC-3 cells (Figure 2A, 3A).

In MCF7 cells the increase in luciferase activity was more evident when the pGL4-Locus1 reporter construct was co-transfected along with a pCMV-AR expression vector, particularly after the treatment with 100 nM DHT (Figure 2B). Interestingly, in these latter experimental conditions, the (G > A) SNP alleles within the putative enhancer region exhibited a different responsiveness with significantly higher luciferase activity detected with the pGL4-Locus1_A construct, (*p* = 0.028, determined by Student's t-test) (Figure 2B). Surprisingly, this SNP-dependent effect was not significant after combined addition of DHT and E2.

On the contrary, in PC-3 cells the Locus 1 did not elicit any enhancer activity (Figure 3B), suggesting that endogenous tissue-specific cofactors are requested for the transcriptional regulation.

When both cell lines cells were co-transfected with the pGL4-Locus2 reporter along with the pCMV-AR expression vector and supplemented with 100 nM DHT, the induction of the reporter was remarkably enhanced, with equal magnitude for both SNP alleles (Figure 2C, 3C). Furthermore, the treatment with E2 led to a moderate increase in transactivation in MCF7 but not PC-3 cells, and the combination of the two hormones showed neither additive nor antagonistic effects for Locus 2 in both cell lines.

We also demonstrated that DHT treatment elicited the same transcriptional effect also at a lower concentration (10^−9^M) (*p* = 0.017; Figure 2D). To further prove the AR-dependent transcriptional activation upon DHT, we treated MCF7 cells over-expressing AR with Casodex (10^−5^M, also known as Bicalutamide), a strong non-steroidal anti-androgenic compound. In the presence of Casodex, the increase in pGL4-Locus 1 responsiveness was significantly lower compared to cells treated with DHT alone (Figure 2D). As additional positive control, cells were co-transfected with pGL4.15-PSA reporter and pCMV-AR plasmid. A high induction of reporter expression was shown when cells were treated with DHT compared to pGL4.15 control vector (Figure S2).

As previously demonstrated by Berger andco-workers [24], the treatment of MCF7 cells with 100 mM DHT led to a relevant increase in endogenous AR protein expression when compared to both EtOH or E2 treatment (Figure 2E). On the contrary, the amount of ER-α protein was relatively unaffected by the treatments. In PC-3 cells the treatment with DHT and E2 led to the same results as in the MCF7 cells in terms of relative changes in AR and ER-α protein levels (both showing lower endogenous levels compared to MCF7 cells) (Figure 3D and 3E).

### Potential gene targets of AR bound enhancers

To identify potential target genes of the selected enhancers, we queried a human tissue dataset for gene transcripts in their surrounding genomic regions and selected four genes around Locus 1 (*PBX1P*, *SHC1*, *DCST2, ADAM15*) and three genes around Locus 2 (*RAB20*, *ING1*, *ARHGEF7*). Although the luciferase assays showed high responsiveness for both enhancer regions, we were not able to detect any increase in the expression of the selected genes in MCF7, with the exception of *ADAM15* in the presence of AR over-expression and upon DHT treatment (see Figure S3A and S3B). The treatment of PC-3 cells with 100 nM DHT for 24 hours led to a considerable increase in endogenous *RAB20*, *ARHGEF7*, and *ING1* expression when compared to EtOH treated cells (Figure S3D). The expression level of *ING1* increased also when the cells were treated with DHT for 16 or 36 hours. Consistent with the luciferase assays, in PC-3 cells genes located near the Locus 1 were only slightly inducible by DHT (*PBXIP1* and *ADAM15*) (Figure S3C). As expected, MCF7 and PC-3 cells showed *KLK3* up-regulation upon DHT treatment (Figure S3A–S3C, inserts). *TFF1* resulted in up-regulation upon E2 treatment consistent with ER-α dependent transactivation in MCF7 cells, where no up-regulation of endogenous *TFF1* was detected in PC-3 cells (mainly expressing ER-β) (Figure S3B–S3D, inserts).

### AR binds Locus 1 and Locus 2 PRRs and rs2242193 impacts on AR recruitment

**Figure 4:**
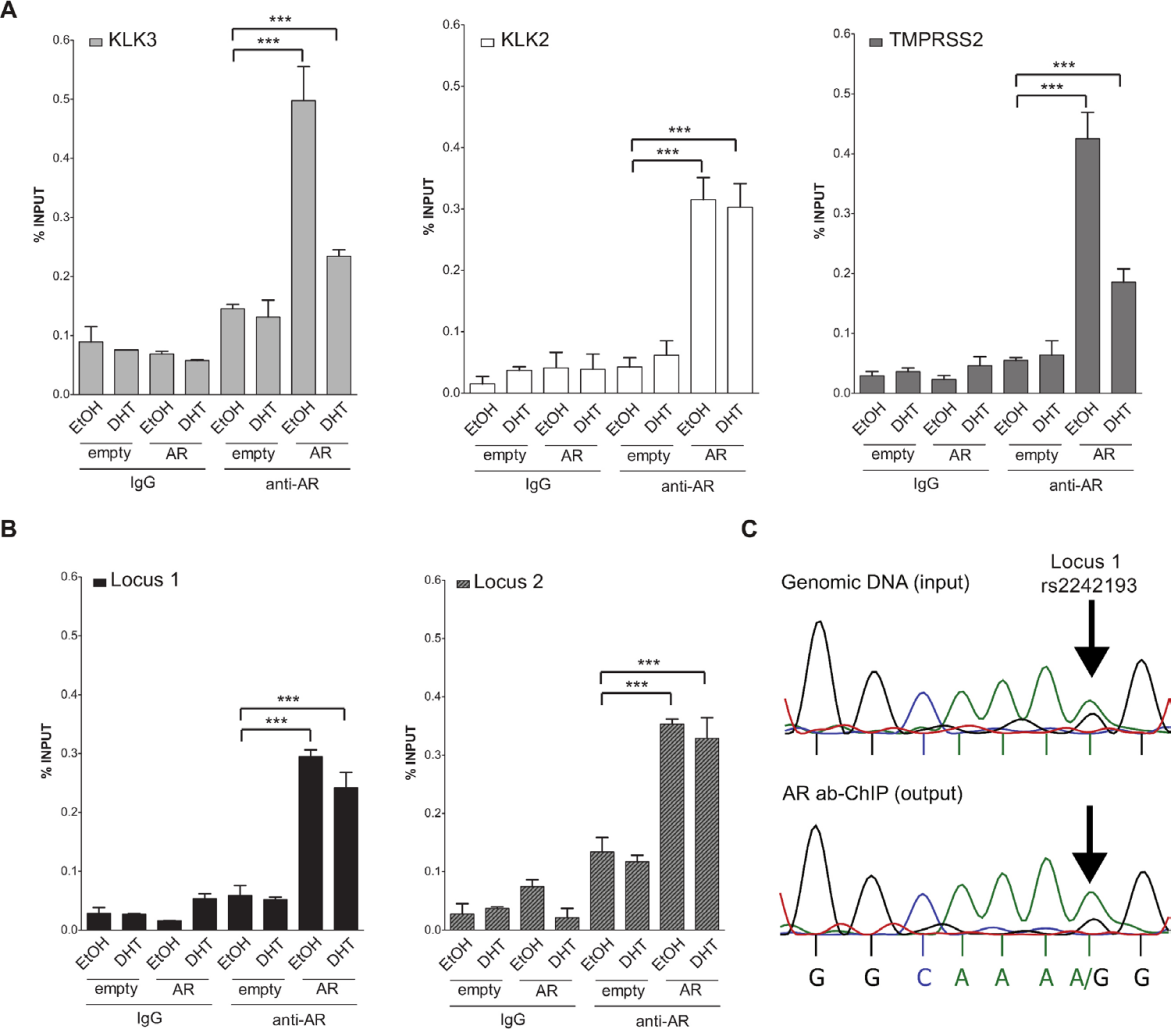
Both locus 1 and locus 2 are directly bound by AR **(A)** A series of ChIP-qPCRs were performed in MCF7 cells (heterozygous for SNP rs2242193 within Locus 2) to determine AR chromatin binding at positive control enhancers -KLK3 (light grey bars), KLK2 (white bars) and TMPRSS2 (dark grey bars)- and **(B)** at Locus 1 and Locus 2 regions (presented respectively as black and grey-patterned bars, respectively). Mean ± s.d. of three technical replicates were plotted. *p < 0.05, **p < 0.01, ***p < 0.005, Student's t-test. **(C)** ChIP analysis from panel **(B)** was followed by standard PCR to amplify Locus 1 region and direct-sequencing was performed to quantify AR recruitment. The specific peaks involving the SNP rs2242193 are highlighted with arrows. Electropherograms showed that AR was preferentially recruited to the A allele of the SNP rs2242193 (*p* < 10^−22^). Input samples from ChIP assay were used as a control.

As both loci were confirmed as transcriptionally responsive to DHT by luciferase assay, we next opted for ChIP assays with AR antibody (or with normal IgG as a control) using MCF7 cells that are heterozygous at rs2242193 in Locus 1, but homozygous for the reference allele at Locus 2. Using quantitative PCR (qPCR), we were able to detect AR binding to both selected loci in MCF7 cells transiently over-expressing AR (Figure 4A, 4B). Occupancy levels at KLK3, KLK2 and TMPRSS2 were measured as positive controls. Moreover, to assess whether AR showed allele-specific DNA binding at rs2242193, we amplified AR-enriched Locus 1 region by standard PCR followed by double-strand direct DNA sequencing analysis. Quantification of the electropherograms showed that the A allele was significantly enriched (*p* < 10^−22^, Fisher test) in chromatin fragments immunoprecipitated with antibody against AR compared to input genomic DNA (Figure 4C and Figure S4A for the biological replicate). Allele-specific PCR confirmed the higher relative occupancy of AR to the A allele (Figure 4C and Figure S4B for qPCR profiles). Collectively, these results showed that AR was preferentially recruited to the A allele of rs2242193. These observations were consistent with the significant increase in luciferase activity obtained with the reporter construct containing the A allele in MCF7 cells (Figure 2B).

## DISCUSSION

Inherited variants and somatic mutations located in intronic or intergenic genomic regions far from any oncogene or tumor suppressor gene may alter cancer susceptibility, influencing distal enhancer elements that regulate the expression of critical target genes [23, 25]. Indeed, the majority of all noncoding GWAS SNPs either lies within a DNase I Hypersensitive Sites or is in complete linkage disequilibrium with SNPs in a nearby DNase I Hypersensitive Sites [26]. Few of them were demonstrated to modulate transcription factor binding, leading to allele specific transcriptional activity. The SNP rs6983267 located within an enhancer modulates the expression of the MYC oncogene [13] and affects a binding site for the Wnt-regulated transcription factor TCF4 [14]; the rs4590952 SNP that resides in a functional p53-binding site regulates the transcription of the KITLG gene [27], and the rs5758550 SNP influences CYP2D6 expression [28]. A recent study showed that androgen receptor binding and transactivation within an androgen response element upstream of the TMPRSS2 gene is affected by a variant, rs8134378, [29]. In the context of tumour genomes, noncoding driver mutations in the TERT promoter were reported in multiple tumor types [30, 31] and nearly additional hundred noncoding driver candidates across the genome were nominated through an integrative study exploiting variants annotations from more than one thousand individuals [23].

In this study we applied a computational approach to select new putative polymorphic regulatory regions (PRRs), defined as regulatory elements spanned by SNPs that may influence the binding of transcription factors. Forty-one SNPs spanning regulatory regions were identified and two PRRs were selected for *in vitro* characterization (on 1q21.3 and 13q34). Towards their functional characterization, we cloned the two PRRs and tested them in gene reporter and ChIP assays separately examining the two SNP alleles. Both PRRs demonstrated enhancer activity and exhibited androgen-responsiveness in at least one cell line. The rs2242193 on 1q21.3 (Locus 1) exhibited allele-specific differences in MCF7 cells, where no enhancer activity was elicited in PC-3 cells. The 13q34 region (Locus 2) showed a modest trend for negative impact of the SNP allele in PC-3 cells, while for highly responsive MCF7 cells no effect was appreciated. These cell specific differences can be related to the expression of endogenous tissue-specific cofactors that are needed for the transcriptional regulation. Specifically, ligand-bound AR translocates to the nucleus, binds to androgen responsive elements (AREs) and modulates gene expression through the induction of chromatin reorganization, epigenetic histone modifications at target genomic loci, and through the recruitment of multiple co-regulator complexes. Proteins that interact with the AR can be divided into three general classes: (i) components of the general transcriptional machinery (e.g. TFIIB and TFIIF [32]; (ii) functionally different proteins with AR co-activating or co-repressing properties (e.g. histone acetyl transferases (HATs), co-activators such as NCOA1 (SRC1, AIB1), NCOA2 (TIF2, SRC2), NCOA3 (SRC3), and co-repressors like SIRT1 and NCOR1 [33–36] and (iii) specific transcription factors that differ from general transcription factors. Some of them interact directly with AR (DAX-1 with the AR Ligand Binding Domain -LBD-) [37] and affect its ability to be recruited at ARE sites without binding directly to the DNA. Other factors such as AP-1 can compete with AR for co-regulators that are present in limited amount within the cell [38]. Alternatively, some transcription factors (e.g., Foxa2 [39]) might bind to DNA sequences allowing cooperation and transcriptional co-regulation of target genes. The dependency of AR on its co-regulators to form a productive transcriptional complex could explain the tissue-selective androgen-dependent gene expression.

By ChIP assay experiments in MCF7 cells, we found that both loci were enriched in chromatin fragments immunoprecipitated with AR antibody and, importantly, detected AR preferentially recruited to the A allele of the SNP rs2242193 (*p*-value < 0.05). This result suggested that the genetic variant rs2242193 might have an impact on the AR recruitment to the chromatin by changing a single base of ARE sequence within the enhancer. In order to verify if the SNPs of interest fall within AR- and/or ER-DNA binding sites we aligned [40] short sequences surrounding the SNPs within Locus 1 and Locus 2 against the ARE (half-site ARE: RGNACR) [41] and the ERE (RGGTCANNNTGASCY) [42] consensus sequences (Figure S5). This comparison indicates that rs2242193 maps to an important AR-DNA contact site (although the site has two additional nonconsensus bases) whereas rs9521825 maps to a less conserved position of a site that is overall a better match to the ARE consensus. Overall this agrees with the luciferase assay results, where the rs2242193 variant A strongly affected the androgen-responsiveness and with the ChIP assay where AR was preferentially recruited to the A allele. The ERE consensus is more conserved compared to the ARE consensus (Figure S5). It has been demonstrated that two nucleotide changes, one in each arm of the palindrome, are sufficient to inhibit ER-α binding [43] and different studies report that cell-specific factors regulate ER transcriptional activation [44, 45]. Moreover, PC-3 cells expresses mainly ER-β rather than ER-α [46]. All together, these observations may explain the luciferase assay results where the E2 treatment led to increased transactivation in MCF7 but not in PC-3 cells and the induction of TFF1 endogenous expression upon E2 treatment in MCF7 cells only.

We posit that AR occupancy at Locus 1 and Locus 2 distal enhancers potentially influenced by the SNP genotype would possibly contribute to AR-dependent modulation of the expression of putative target genes and performed qPCR analysis to evaluate the endogenous expression levels of genes located in the proximity of the PRRs and their relative abundance in response to ectopic AR and/or DHT treatment. Based on RNA-seq data, we selected four (*PBX1P*, *SHC1*, *DCST2, ADAM15*) and three (*RAB20*, *ING1*, *ARHGEF7*) genes in the surroundings of Locus 1 and Locus 2, respectively. The treatment of PC-3 over-expressing AR with 100 nM DHT led to a relevant increase in endogenous *RAB20*, *ARHGEF7*, and *ING1* expression. Consistent with the luciferase assays, no gene located near the Locus 1 was induced by DHT. Albeit indirectly, these results suggest that the transcriptional activity of the aforementioned genes are under the control of this enhancer. Recent studies suggested a role for a number of RAB proteins in human cancers both as activators and inhibitors in tumor progression. In particular, RAB20 expression is increased in exocrine pancreatic adenocarcinomas [47] and in breast cancer [48]. Moreover, RAB20 amplification was also correlated with high-grade dysplastic colorectal adenomas [49]. Over-expression of *ING1* can induce cell cycle arrest and apoptosis [50], enhance expression of the Bax gene and was reported to alter mitochondrial membrane potential in a p53-dependent manner [51]. INGs also function in histone acetylation [52, 53] acting as stoichiometric partner for HAT and HDAC complexes [54]. In MCF7 cells where AR was over-expressed, only *ADAM15* gene expression increased significantly upon treatment with DHT (Figure S3). These results underline the strong variability of AR-induced responsiveness in different cell lines previously reported also among cell lines of the same tissue derivation; for instance, only 11% of the androgen-responsive genes reported in HPr-1AR cells [41] were consistently activated by AR in LNCaP cells [55, 56]. The metalloproteinase *ADAM15* is a multi-domain disintegrin protease that maps to a region of documented amplification associated with the metastatic progression of human cancers, including prostate, breast, ovarian, colon, and melanoma [57–59]. *ADAM15* mRNA and protein levels are increased in prostate cancer and its expression is significantly increased during metastatic progression. *ADAM15* may down-regulate adhesion of tumor cells to the extracellular matrix, reduce cell-cell adhesion, and promote metastasis through the activity of its disintegrin and metalloproteinase domains [60]. Given its diverse functions, ADAM15 may represent a pivotal regulatory component of tumor progression and an important target for therapeutic intervention.

Whereas our approach to the study of PRRs can in principle be pursued for any transcription factor of interest, we reasoned that AR and ER in the context of prostate and breast cancer cells would provide an excellent proof of principle as both diseases are characterized by a prominent hereditary component and as alternative roles for AR and ER have been suggested for both diseases. A significant number of poorly differentiated breast carcinomas are ER-negative but AR-positive suggesting AR as a useful marker for the further refinement of breast cancer subtype classification and as an independent prognostic factor and therapeutic target for the triple-negative breast cancers [61, 62]. On the other hand, although the AR remains the major target for prostate cancer prevention and treatment, ER is also involved in prostate cancer development and tumour progression. ER-α signalling potentiates the carcinogenic effects of androgens on the prostatic epithelium [22] and levels of E2 that increase with age may contribute to prostate cancer risk [63]. Epidemiologic studies suggested that African Americans, at the highest risk of prostate cancer, show the highest levels of estrogens [64]. Moreover, there is evidence that the treatment of the high-grade prostatic intraepithelial neoplasia with the antiestrogen toremifene leads to decreased cumulative risk of prostate cancer diagnosis [65]. Where the functional significance of the potential co-occupancy by AR and ER at enhancer sites (see Table S3) and the impact of androgen and estrogen relative levels remain to be established, we hypothesize that AR/ER bound PRRs can play a cell specific role in AR/ER-dependent modulation of target genes based upon the individual's genotype and overall potentially contribute to carcinogenesis mechanisms.

Several studies have noted that GWAS associated risk SNPs map to enhancers at higher than random rates. Altogether, our results show that the unbiased genome-wide search for PRRs is an efficient methodology to discover new functional cis-elements relevant to hormone driven diseases and beyond by providing experimental evidence for selected variants mapping to regulatory regions. Further studies will extend on the understanding of specific disease mechanisms and on the design of strategies for individuals' risk assessment and treatment to eventually improve the processes of drug selection and dosing. The broad collection of more than 130 TFs analyzed in this study identified PRRs of potential interest for the research community towards the understanding of rare and common variants in cis-regulatory sequences.

## MATERIALS AND METHODS

### Selection of responsive regulatory regions

ChIP-Seq ENCODE data were queried for 17 cell-lines selected based on H3K4m1 and H3K4m3 data availability. The set includes the following; GM12878, H1-hESC, Hela-S3, HepG2, HMEC, HSMM, HSMMtube, HUVEC, K562, Monocytes-CD14+, NHA, NHDF-Ad, NHEK, NHLF, Osteobl, Dnd41, LNCaP. *Consensus regulatory regions* were determined for enhancer/promoter pattern (H3K4me1/H3K4me1+H3K4me3) and for additional activation and open chromatin markers (H3K9ac, H3K27ac, DNase-seq and FAIRE-seq). Specifically, for each marker the consensus was generated as the merge of all the peak regions that were detected in at least two cell lines as follow: (i) retain a peak if it overlaps for at least 50% of its length with a peak in second cell line; (ii) concatenate all the peaks obtained from step i; (iii) sort and merge retained peak regions. ENCODE data as per January 2013.

For each ENCODE TF (*N* = 134) and for ER-α [66] and AR [67] ChIP-Seq data, a list of regions of interest was compiled based on 3,568,312 SNPs from dbSNP138 (merged list across populations) upon selection of binding peak regions across cell lines (as described above). To exploit paired genotype/phenotype data from sequencing experiments and high density oligonucleotide data [68, 69] SNPs represented on the Affymetrix SNP 6.0 (N SNP = 924,395) were tracked through the study and prioritized for functional validation.

### Cell lines and compounds

Breast adenocarcinoma-derived MCF7 cell line (positive both for Estrogen and Androgen Receptors) and Prostate Cancer-derived PC-3 cell line (positive for Estrogen Receptor alpha and negative for Androgen Receptor) were purchased from ATCC (American Type Culture Collection, LGC Standards, Milan, Italy).

MCF7 and PC-3 cell lines were maintained respectively in Dulbecco's Modified Eagle Medium (DMEM) or Roswell Park Memorial Institute medium (RPMI) (Gibco, Life Technologies, Milan, Italy) that were supplemented with 10% Fetal Bovine Serum (FBS), 100 units/ml penicillin, 100 μg/ml streptomycin, and 2 mM L-Glutamine. Cells were grown in humidified atmosphere at 37°C with 5% CO_2_ in a cell culture incubator.

Sex hormone depletion (androgens and estrogens), prior to dihydrotestosterone (DHT) (Sigma-Aldrich, Milan, Italy) and 17β-estradiol (E2, Sigma-Aldrich, Milan, Italy) treatments, was achieved by growing the cells in medium without Phenol Red (Euroclone, Celbio, Milan, Italy), supplemented with 10% charcoal/dextran treated FBS (Hyclone, Celbio, Milan, Italy) for 48 hours.

### Plasmids and dual iuciferase assay

The genomic sequence of the selected responsive regulatory regions was amplified using 5PRIME MasterTaq kit (5PRIME, Milan, Italy) in order to get an adequate replication fidelity on genomic DNA available obtained from HUVEC cells (Human Umbilical Vein Endothelial Cells from a male donor). Primers were selected with the Primer-BLAST web tool (http://www.ncbi.nlm.nih.gov/tools/primer-blast/) (Table S1). PCR products for the two selected loci of interest (fragment sizes of 1009 bp (Locus 1) and 1205 bp (Locus 2)) were cloned upstream the luciferase cDNA into pGL4.26 vector (Promega, Milan, Italy) using KpnI and XhoI restriction endonucleases and T4 DNA Ligase (New England Biolabs, Euroclone). Constructs harboring the alternative allele for rs2242193 and rs9521825 were created with the GeneArt®eSite-Directed Mutagenesis System (InVitrogen, Life Technologies) according to the manufacturer's instructions. (Table S1). The correct insertion of the desired genomic sequence and SNP alleles in pGL4.26 was checked by direct DNA cycle sequencing (BMR Genomics, Padua, Italy). All plasmids were purified from XL1-Blue *E. coli* bacterial cells using the PureYield™ Plasmid Midiprep system protocol (Promega). The day before transfection, MCF7 and PC-3 cells (6 or 8 × 10^4^ cells, respectively) were seeded in 24 well plates. Cells were co-transfected using the FugeneHD reagent (Promega) or TransIT-LT1 reagent (Mirus, TemaRicerca, Bologna, Italy) with pGL4.26-derived reporter vectors (350 ng) and pCMV-AR24Q expression vector (100 ng, to over-express AR). The total DNA amount was kept constant by the addition of the empty vector pCMV-NeoBam. All transfections were normalized for efficiency using 50 ng of the pRL-SV40 vector (Promega). Twenty-four hours after transfection, cells were treated with either 10^−7^ M DHT, or 10^−7^ M E2 or the combination of the two compounds for at least 16 h, diluted in hormone- and serum-free medium. To determine whether DHT elicited the same transcriptional effect also at a lower concentration, MCF7 cells were treated with 10^−9^ M DHT. Moreover, to verify the AR-dependent transactivation of the selected regulatory regions, we used the non-steroidal anti-androgen compound Bicalutamide (10^−5^ M, also known as Casodex, Sigma). Forth-eight hours after transfection, cells were lysed using Passive Lysis Buffer 1 × (Promega) and firefly and Renilla luciferase activities were measured as previously described [70] with Dual-Luciferase Reporter Assay (Promega) using the Infinite M200 multi-plate reader (Tecan, Milan, Italy).

### Real-time qPCR

Total RNA was extracted from MCF7 (seeded as 1.5–3 × 10^5^ cells in a 6 well plate) and PC-3 (2.5–3.5 × 10^5^) cells transfected with AR or empty vectors (2.5 μg per well) and treated with DHT, E2 or the combination of the two compounds using the RNeasy kit (Qiagen, Milan, Italy) according to the manufacturer's instructions.

Two μg of total RNA was converted in cDNA using the RevertAid First Strand cDNA Synthesis Kit and the M-MuLV reverse transcriptase enzyme (ThermoFisher Scientific, Milan, Italy). Then, quantitative PCR reactions in real-time were performed as previously described [71] using KAPA SYBR® FAST Universal 2 × qPCR Master Mix (Kapa Biosystems, Resnova, Ancona, Italy) using the CFX384 or CFX96 Detection Systems (BioRad, Milan, Italy). Primer specificity and efficiency was tested with standard procedures. Analysis of relative mRNA expression was performed using the ΔΔCt method with GAPDH (Glyceraldehyde 3-phosphate dehydrogenase) and B2M (beta-2 microglobulin) as reference genes. Canonical targets for ER and AR, TFF1 and KLK3 respectively, were used as positive controls. qPCR analysis was also performed to evaluate the endogenous expression levels of genes located in the proximity of the region of interest (Table S1).

### Western blot

Endogenous as well as ectopically expressed soluble proteins were extracted from 6 well plates with 100 μl of ice-cold RIPA (Radio Imuuno-Precipiatation Assay) lysis buffer supplemented with protease inhibitor cocktail (Roche, Milan, Italy). Equal amounts of proteins (50 μg) were resolved by 12% SDS-PAGE and transferred to nitrocellulose membranes using the semi-dry iBlot Transfer System (InVitrogen, Life Technologies). Membranes were blocked with 5% non-fat skim milk dissolved in PBS-T and incubated at 4°C overnight with primary anti-AR (clone #: D6F11, Cell Signaling, Milan, Italy) (1:1000) and –ER-α (clone #: E115, Millipore, Milan, Italy) (1:500) antibodies diluted in 1% milk in PBS-T. Detection of GAPDH levels (clone #: 6C5, Santa Cruz Biotechnology, Milan, Italy) or β-Tubulin (clone #: 3F3-G2, Santa Cruz) served as loading control. Membranes were then incubated with secondary goat anti-mouse (1:10000) or goat anti-rabbit antibodies (Sigma-Aldrich) (1:12000) for 1 h at room temperature. Detection was achieved using the ECL Select detection reagent (Amersham, GE Health Care, Milan, Italy) with the ChemiDoc XRS+ System (BioRad).

### Chromatin immunoprecipitation (ChIP) assay

MCF7 cells were maintained into 150 mm Petri dishes in RPMI medium without Phenol Red, supplemented with 10% charcoal/dextran treated FBS. Two days after, cells were transfected with pCMV-AR24Q expression vector or the pCMV-NeoBam empty vector. Then, cells were treated with DHT (10^−7^M) or EtOH. After 16 hours of treatment, cells were harvested and ChIP assays were performed as previously described [72, 73]. Briefly, protein-DNA complexes were cross-linked by the addition of 1% formaldehyde that was quenched with 0.125 M Glycine. Chromatin was shared with cycles of sonication using a Misonix S-4000 sonicator (Misonix, Newtown, CT, USA) to generate fragments with an average size of 150–400 bp. Small aliquots of sample (10%) were used as input DNA. Chromatin immunoprecipitation was performed with anti-AR antibody (ChIPAb+ androgen receptor Assay Kit, Millipore) using the Magna ChIP G kit (Upstate, Millipore) according to the manufacturer's instructions. We next performed real-time quantitative PCR with Sybr Green as described above, followed by cycle sequencing analysis (BMR Genomics). Known AR target genes (*KLK3*, *KLK2*, *TMPRSS2*) were selected as positive control. Amplification of the selected regulatory regions was also performed (Table S1). AR specific recruitment was calculated as % of input signals respect to the EtOH treated cells according to the ΔCt method. ChIP analysis was followed by PCR to amplify and direct-sequencing Locus 1 region (both forward and reverse strand). The area under the peak corresponding to A and G allele of the SNP was integrated and quantified using the ImageJ software. The area of A allele (A) was normalized to the area of allele G (G) using the following formula: (A/(A + G))*100. The difference between the input and output DNA was compared using the Fisher test. Allelic specific PCR was performed to further support the impact of rs2242193 on AR occupancy, starting from DNA obtained by ChIP experiments in MCF7 cells that are heterozygous for this SNP (Table S1).

## SUPPLEMENTARY FIGURES AND TABLES






