# Supplementary material for: In silico identification and functional validation of allele-dependent AR enhancers
Source: Oncotarget. 2015 Feb 27;6(7):4816–28. doi: 10.18632/oncotarget.3019 (PMC4467117; doi:10.18632/oncotarget.3019)
Supplement: Supplementary file 1 [file oncotarget-06-4816-s001.pdf]

SUPPLEMENTARY FIGURES AND TABLES

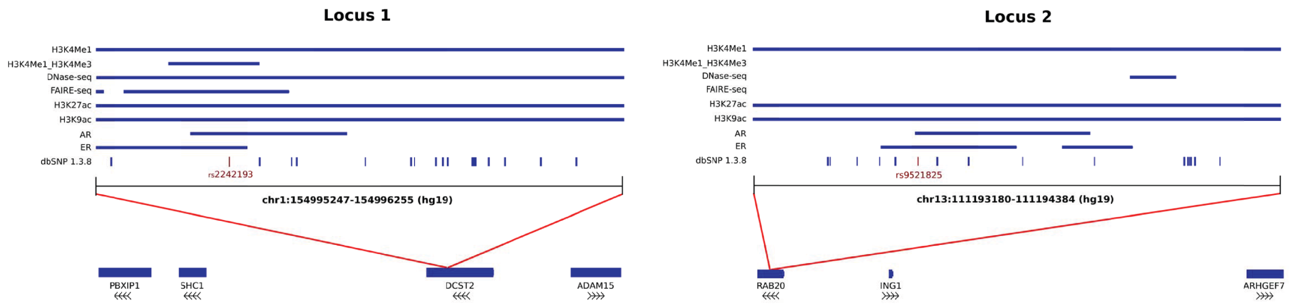

Supplementary Figure S1: Schematic description of Locus 1 (left) and Locus 2 (right) genomic regions. Activation histone marks peaks, open chromatin peaks and AR/ER binding regions, along with SNP loci are depicted; SNPs of interest are highlighted. The bottom part of the two panels provides a broader view of the two locus location along with surrounding genes of interest in flanking regions of 1M bp.

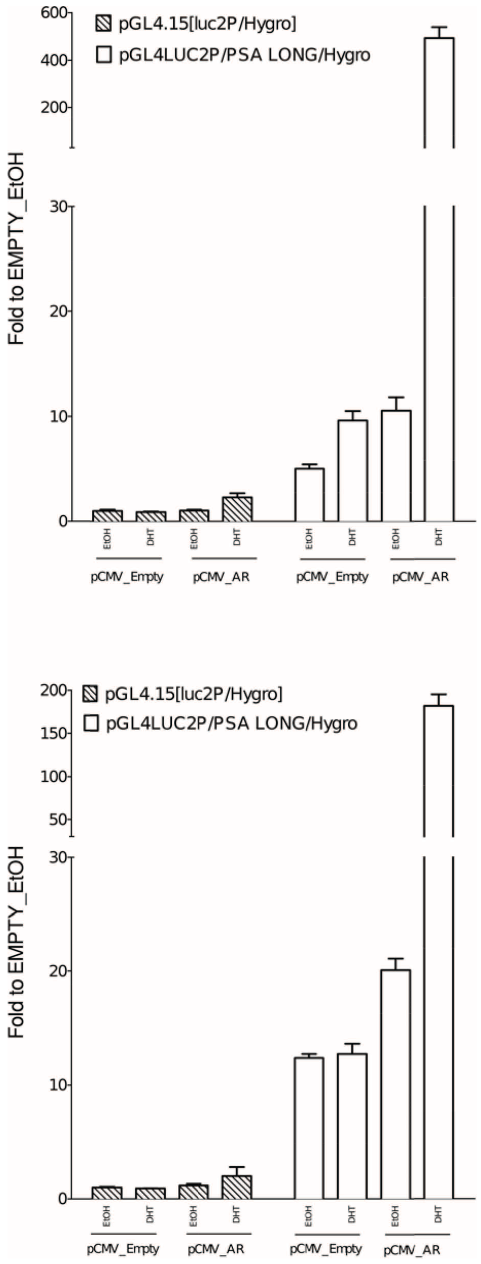

Supplementary Figure S2: As positive control of luciferase assay, MCF7 and PC-3 cells were co-transfected with pGL4.15-PSA reporter and pCMV-AR plasmid. A high induction of reporter expression was detected when cells were treated with DHT compared to pGL4.15 control vector.

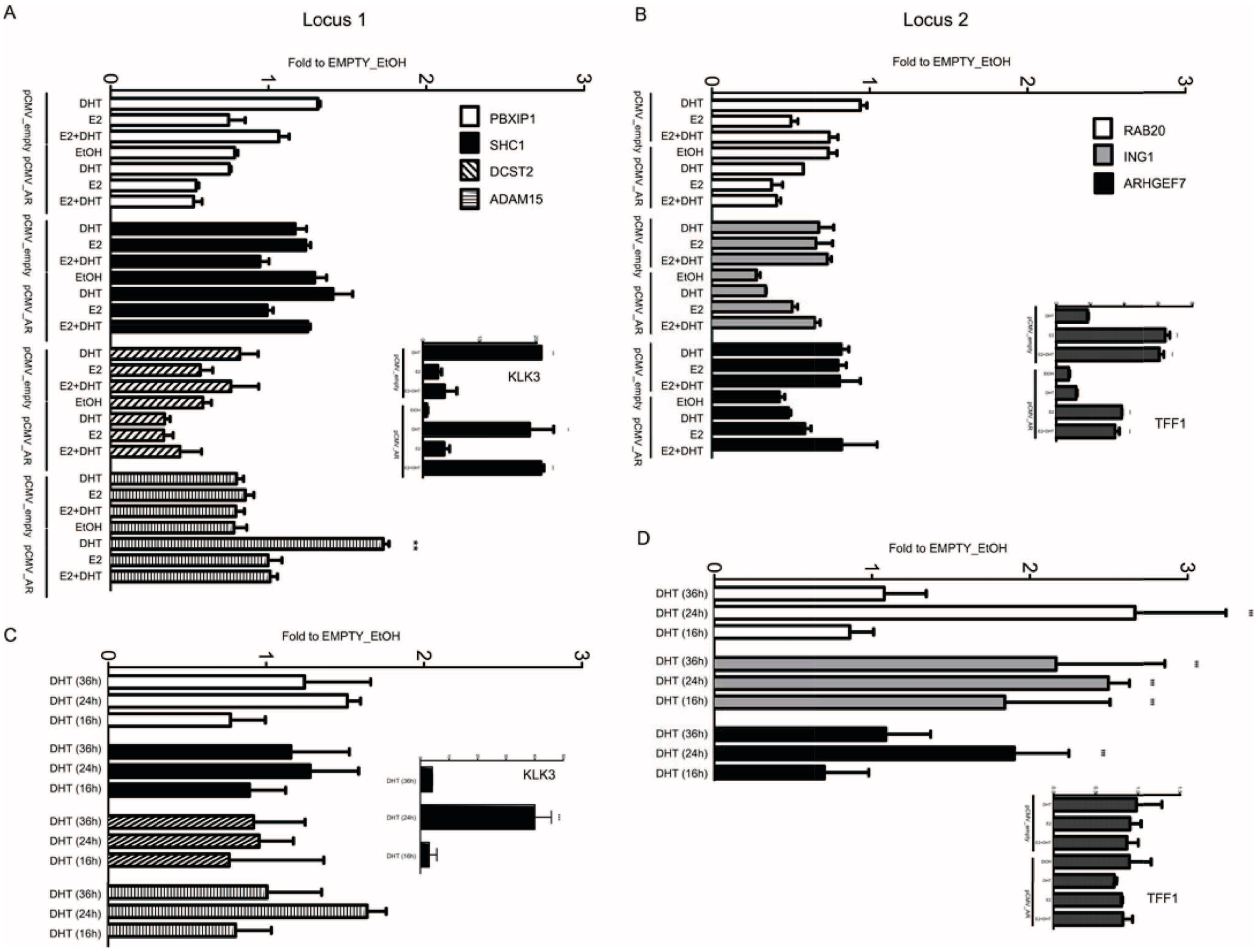

Supplementary Figure S3: The expression of different genes in the proximity of locus 1 or locus 2 can be positively regulated by AR. The endogenous expression levels of genes located in the proximity of the enhancer locus were evaluated in MCF7 cell line transiently transfected with AR expression vectors and treated with E2, DHT and the combination of the two compounds **(A, B)** and in PC-3 cell line **(C, D)**, transiently transfected with AR expression vectors and treated with DHT. The data are shown as fold to the condition transfected with pCMV_EMPTY and treated with EtOH. We select four genes near the locus 1 (PBX1P, SHC1, DCST2, ADAM15) and three genes near the locus 2 (RAB20, ING1, ARHGEF7). Canonical targets for AR (KLK3), or for ER-α (TFF1), were used as positive controls (indicated in the inserts). The treatment with 100 mM DHT led to a relevant increase in endogenous RAB20, ING1, and ARHGEF7 expression in PC-3 cells, and in ADAM15 expression in MCF7 cells. $*p < 0.05$, $**p < 0.01$, $***p < 0.005$, Student's *t*-test.

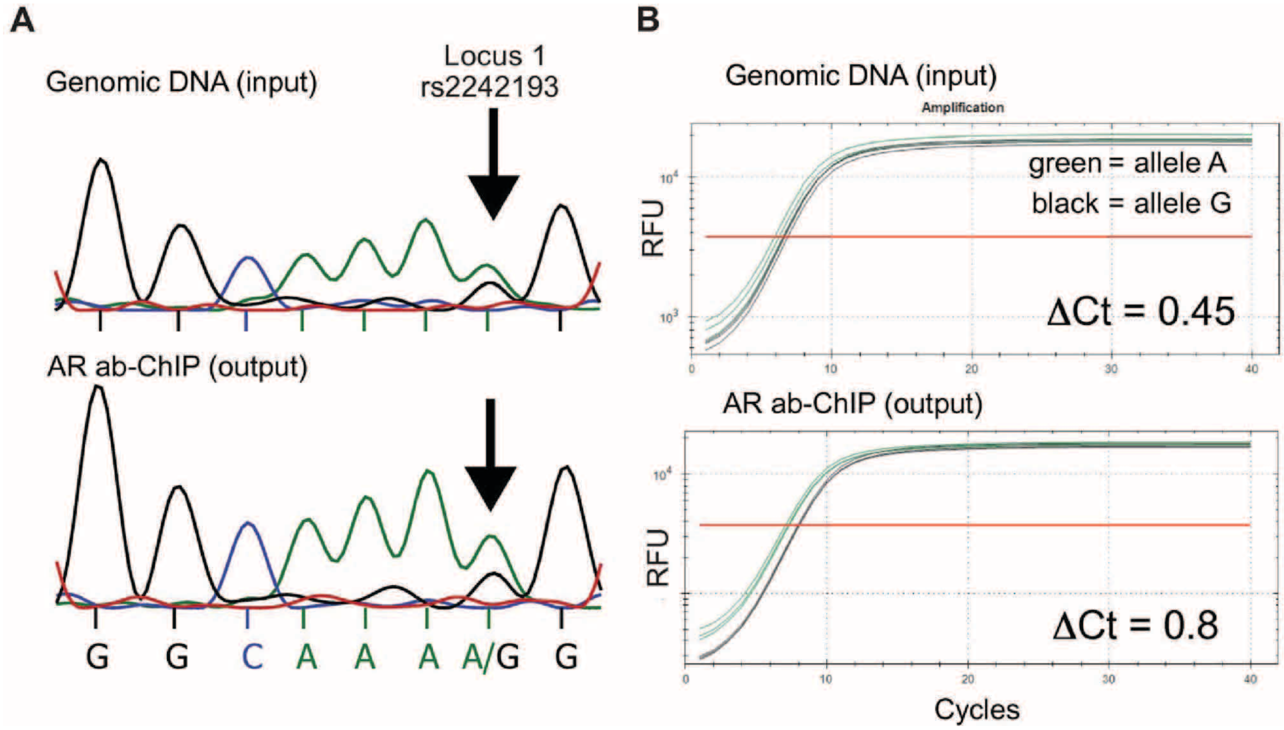

Supplementary Figure S4: (A) Biological replicate of the experiment described in figure 4B. Chip was followed by direct-sequencing to quantify AR recruitment to the locus 1. Electropherograms showed that AR was preferentially recruited to the A allele of the SNP rs2242193 (highlighted with arrows). **(B)** ASO-PCR was performed to selectively amplify the G or the A allele of the rs2242193 SNP in the DNA samples from ChIP assay (primers are listed in Table S1). In order to calculate the initial amount of each allele we calculate the ΔCt between the G allele and the A allele. Our data showed a ΔCt of 0.80 in the output and of 0.45 in the input, confirming the higher relative occupancy of AR to the A allele.

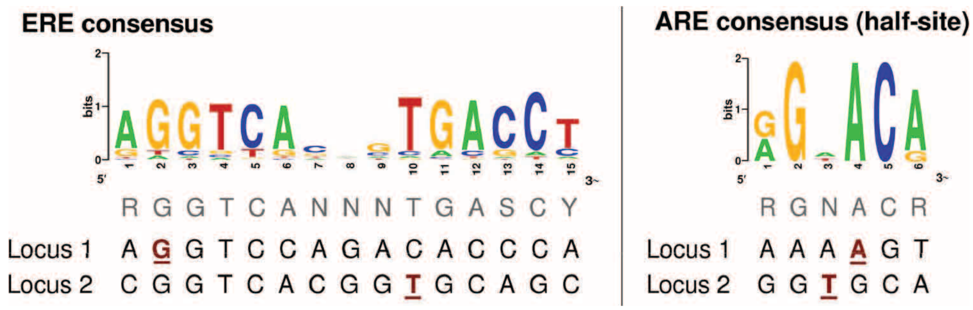

Supplementary Figure S5: Sequences surrounding the study SNPs (underlined) within Locus 1 and Locus 2 were aligned against the ERE and the ARE consensus sequence based on the literature ((1) and (2), respectively). Letter heights represent the degree of base conservation at that position.

Supplementary Table S1: Sequences of primers used in this study

Name	5′-3′
**Cloning_primers**	
Locus_1_cloning_F	aaGGTACCGCTAGCCCAGCTCATGTATCACCTCTTCTAT
Locus_1_cloning_R	aaAGATCTCTCGAGATATGACAATCCACTGTGGTCCTTC
Locus_2_cloning_F	aaGGTACCGAGCTCATCTCTCTCACGCAAAATAGTGTCT
Locus_2_cloning_R	aaAGATCTCTCGAGCTTATTATTTGGCAGTTCCTTCCCC
**Mutagenic_primers**	
Locus_1_MUT_F	CTGTTTCAAGCCCTGGCAAAAGTCCAGACACCCAGGAAGGC
Locus_1_MUT_R	GCCTTCCTGGGTGTCTGGACTTTTGCCAGGGCTTGAAACAG
Locus_2_MUT_F	CCACCTGGTCACGGTCACGGTGCAGCAACAGATTCCTCAAG
Locus_2_MUT_R	CTTGAGGAATCTGTTGCTGCACCGTGACCGTGACCAGGTGG
**real_time_primers**	
B2M_ qPCR_F	AGGCTATCCAGCGTACTCCA
B2M_ qPCR_ R	ATGGATGAAACCCAGACACA
GAPDH_ qPCR_F	TCCAAAATCAAGTGGGGCGA
GAPDH_ qPCR_R	AGTAGAGGCAGGGATGATGT
DCST2_qPCR_F	TACTGCCTCACTTGCTTCCG
DCST2_qPCR_R	TAGTGGCGGAGGATGTCTCA
RAB20_qPCR_F	CCCGAGTGTCCTATGCCTTC
RAB20_qPCR_R	GTCCACTTTGTTCCCCACGA
PBXIP1_qPCR_F	TGTCATCGTCACTGCTGGA
PBXIP1_qPCR_R	CAGACACACAGGACCTGGAA
SHC1_qPCR_F	CTGACACTTTCAAAGCGGTG
SHC1_qPCR_R	GTATGTGCTCACTGGCTTGC
ADAM15_qPCR_F	GGGCACAGGAATGTCGAAG
ADAM15_qPCR_R	CCAGCCCTCCTCACAGTAG
ARHGEF7_qPCR_F	AGGTCACGTCTGTGGGAAAC
ARHGEF7_qPCR_R	GTGCTTGCTGGACGGAGT
ING1_qPCR_F	TCTGTCTCGCGACTGAAGC
ING1_qPCR_R	GCCTAGGCTGCTGGGAGT
**ChIP_assay_primers**	
KLK3_ChIP_F	ATACTGGGACAACTTGCAAACCT
KLK3_ChIP_R	CAGGCTTGCTTACTGTCCTAGATAA
KLK2_ChIP_F	GGTTGAAAGCAGACCTACTCTGG
KLK2_ChIP_R	AGATCTAGGTTTGCTTACTGCCTTAG
TMPRSS2_ChIP_F	GTGGCCCACCACTTCCTCAC
TMPRSS2_ChIP_R	CACACAGCAAGGCAGAGGACA

(*Continued*)

Locus_1_ChIP_F	TCGATGTCACTCTCCCCAAG
Locus_1_ChIP_R	CAGGAAGCAGGAGTTGGCA
Locus_2_ChIP_F	TTCACACCTCACCACTCTCC
Locus_2_ChIP_R	GACACTTCGTTCCCATGTGC
**ASO-PCR_primers**	
Locus_1_ChIP_F	TCGATGTCACTCTCCCCAAG
ASO-PCR_Locus1_G_R	CCTTCCTGGGTGTCTGGACC
ASO-PCR_Locus1_A_R	CCTTCCTGGGTGTCTGGACT

Supplementary Table S2: Characteristics of SNPS in PRRs of interest selected for functional characterization (NCBI Variation Database (dbSNP)

**rs2242193**	**Population**	**Genotype detail**		
		C/C	C/T	T/T
	HapMap-CEU	0.372	0.504	0.124
	HapMap-HCB		0.186	0.814
	HapMap-JPT	0.012	0.140	0.849
	HapMap-YRI	0.265	0.451	0.283
**rs9521825**	**Population**	**Genotype detail**		
		C/C	C/T	T/T
	HapMap-CEU	0.566	0.398	0.035
	HapMap-HCB	0.465	0.465	0.070
	HapMap-JPT	0.477	0.395	0.128
	HapMap-YRI	0.726	0.257	0.018

Supplementary Table S3A: Complete lists of SNPs (CEU) within consensus regulatory regions bound by all considered TFs

Supplementary Table S3B: Complete lists of SNPs (all) within consensus regulatory regions bound by all considered TFs

REFERENCES

1. Shu FJ, Sidell N, Yang D, Kallen CB. The tri-nucleotide spacer sequence between estrogen response element half-sites is conserved and modulates ERalpha-mediated transcriptional responses. The Journal of steroid biochemistry and molecular biology. 2010; 120:172–179.
2. Bolton EC, So AY, Chaivorapol C, Haqq CM, Li H, Y amamoto KR. Cell- and gene-specific regulation of primary target genes by the androgen receptor. Genes & development. 2007; 21:2005–2017.
